# *Bacillus* sp. JR3 esterase LipJ: A new mesophilic enzyme showing traces of a thermophilic past

**DOI:** 10.1371/journal.pone.0181029

**Published:** 2017-07-25

**Authors:** Judit Ribera, Mónica Estupiñán, Alba Fuentes, Amanda Fillat, Josefina Martínez, Pilar Diaz

**Affiliations:** 1 Department of Genetics, Microbiology and Statistics, Faculty of Biology, University of Barcelona, Av. Diagonal 643, Barcelona, Spain; 2 Institute of Nanoscience and Nanotechnology (IN2UB), University of Barcelona, Av. Diagonal 643, Barcelona, Spain; Karl-Franzens-Universitat Graz, AUSTRIA

## Abstract

A search for extremophile enzymes from ancient volcanic soils in El Hierro Island (Canary Islands, Spain) allowed isolation of a microbial sporulated strain collection from which several enzymatic activities were tested. Isolates were obtained after sample cultivation under several conditions of nutrient contents and temperature. Among the bacterial isolates, supernatants from the strain designated JR3 displayed high esterase activity at temperatures ranging from 30 to 100°C, suggesting the presence of at least a hyper-thermophilic extracellular lipase. Sequence alignment of known thermophilic lipases allowed design of degenerated consensus primers for amplification and cloning of the corresponding lipase, named LipJ. However, the cloned enzyme displayed maximum activity at 30°C and pH 7, showing a different profile from that observed in supernatants of the parental strain. Sequence analysis of the cloned protein showed a pentapeptide motif -GHSMG- distinct from that of thermophilic lipases, and much closer to that of esterases. Nevertheless, the 3D structural model of LipJ displayed the same folding as that of thermophilic lipases, suggesting a common evolutionary origin. A phylogenetic study confirmed this possibility, positioning LipJ as a new member of the thermophilic family of bacterial lipases I.5. However, LipJ clusters in a clade close but separated from that of *Geobacillus* sp. thermophilic lipases. Comprehensive analysis of the cloned enzyme suggests a common origin of LipJ and other bacterial thermophilic lipases, and highlights the most probable divergent evolutionary pathway followed by LipJ, which during the harsh past times would have probably been a thermophilic enzyme, having lost these properties when the environment changed to more benign conditions.

## Introduction

Lipolytic enzymes (EC 3.1.1.-), widely distributed in nature, are a diverse group of hydrolases catalysing the cleavage or formation of ester bonds [1,2]. Grouped under the general term of lipases, lipolytic enzymes include “true” lipases (EC 3.1.1.3, triacylglycerol hydrolases) and esterases (EC 3.1.1.1, carboxyl ester hydrolases), which differ in both, their kinetics and chain-length substrate preferences [1,2]. Esterases are active on short-chain length esters partially soluble in water, and lipases have optimal activity towards long-chain triacylglycerides, not soluble in aqueous environments [2]. Therefore, esterases display a typical Michaelis-Menten behaviour, whereas many lipases show interfacial activation when lipids reach an equilibrium between the monomeric, micellar and emulsified states [2]. Although the physiological role of many microbial lipases remains still unclear, in bacteria most esterases are intracellular enzymes involved in lipid metabolism and turnover [3,4]. On the contrary, most bacterial true lipases are secreted enzymes and perform their role extracellularly, probably by procuring nutrients to the cell or by executing synthesis reactions aimed at releasing modified lipid compounds that could act as extracellular cell communication signals, or have a role in detoxification of biocides [3–6].

The 3D structure of both, lipases and esterases, consists on a defined alternation of α-helices and β-sheets forming the characteristic α/β–fold also found in other hydrolases [6–8]. Their active site is constituted by a catalytic triad containing a serine as the catalytic residue, an aspartic or glutamic acid, and a histidine, usually being the nucleophile serine embedded in the GX**S**XG consensus pentapeptide [2,9]. Bacterial lipolytic enzymes were initially classified into eight families [10], with successive revisions up to more than 16 families described nowadays [9,11,12]. These classifications are based on amino acid sequence homology and the presence of conserved motifs, although more recently, certain structural features and phylogenetic analysis have come to complement this classification [9–12].

During the last years the interest generated by lipases has grown exponentially due to both, their broad array of substrate specificity and their versatility in catalysed reactions [13–16]. Therefore, these enzymes are considered powerful biocatalysts with many biotechnological applications such as food technology, detergent formulation, flavour and drug production, or in the synthesis of optically pure compounds, among other uses in fine chemistry [2,13,15–18]. Moreover, lipases do not usually require cofactors, are quite robust and stable, and can be active in organic solvents [1,18–20]. However, not all lipolytic enzymes bear the properties required for a defined biotechnological process, and availability of new catalysts with striking properties is still a challenge [21,22]. For those reactions requiring harsh conditions like high temperature or extreme pH, different strategies can be followed. Among them, microbial isolation from natural environments is one of the most used strategies to discover new enzymes which can fit the conditions required by the industrial processes [23–25]. This is the case for certain environments that supported high temperatures or extreme conditions in old times. Therefore, ancient volcanic soils from Laurel forest (Laurisilva) of El Hierro Island (Canary Islands, Spain) are a good example of such environments, as they suffered from harsh conditions in the past, when the volcanic island appeared approx. 1 million years ago, but evolved to acquire milder conditions nowadays. Evolution of the volcanic island from an extreme environment to its present benign state, a cloudy forest with plenty of organic matter, endemic vegetation and mild temperatures due to the north-east trade winds and the Föhn effect, is an exceptional niche for isolation of undescribed strains with reminiscent extreme biocatalysts. Therefore, a search for new strains was performed, focussing our interest on lipolytic enzymes from sporulated bacteria. Several microbial strains were isolated from an ancient volcanic soil sample, and their catalytic activities analysed. Strain JR3, displaying extracellular lipolytic activity up to 100°C was selected for further prospection and characterization of novel thermophilic lipases. The mixed molecular and biochemical properties of the cloned enzyme LipJ are described and discussed here.

## Materials and methods

### Strains, plasmids and growth conditions

A collection of sporulated strains (Table 1) was isolated from an ancient volcanic soil, now a Laurel forest at El Hierro Island (coordinates 27.735163–17.993231; sampling performed in a public area of the municipality of El Pinar, with permission of the National Institute of Geography of the Canary Islands, Spain), using the conditions stated in Table 1 and following a previously described isolation protocol with modifications [25]. Microorganisms were extracted by suspending 1 g of soil in 10 mL Ringer ¼. After 10 min vigorous stirring and additional sedimentation, aliquots of the upper liquid phase were collected and used for isolation of aerobic and facultative anaerobic spore-forming bacteria by means of serial dilutions in Ringer 1/4. 0.2 mL of each dilution were spread on Luria-Bertani medium (LB) agar plates and incubated at 30°C and 55°C for 1 to 7 days. Those colonies displaying different morphological properties were isolated, their Gram and spore stain performed, and their hydrolytic activity on several substrates at the optimum growth temperature was analysed on Trypticase Soy Agar (TSA) (Pronadisa) plates supplemented with sterile skimmed milk (1% v/v, Sharlau), LB agar plates supplemented with carboxymethyl cellulose (CMC) (0.5% w/v, Sigma), CeNAN (ASDA Micro) supplemented with olive oil (1% w/v, Carbonell) or tributyrine (1% w/v, Sigma) emulsified with 0.1% arabic gum (w/v, Sigma) and 0.0002% Rhodamine B (v/v, Sigma) [26], and incubated at the isolation temperature of each strain, as previously described [25]. Strain *Paenibacillus barcinonensis* BP-23 (CECT 7022) [27] was used as hydrolytic positive control. Pure cultures of each newly isolated strain were maintained at 4°C and stored in glycerol stocks at −80°C.

**Table 1:** Strains isolated from a forest at the volcanic El Hierro Island (Canary Islands, Spain). Activity assays were performed at 30°C on LB-agar plates supplemented with either milk (protease), carboxymethylcellulose (cellulose), olive oil (lipase) or tributyrine (esterase). According to the 16S rDNA sequence and physiological tests, strain JR3 was identified as *Bacillus* sp. JR3, close but not identical to *B*. *cereus*.

Strain	Milk (protease)	Carboxymethylcellulose (cellulase)	Olive oil (lipase)	Tributyrine (esterase)
**JR-1**	**++**	**+++**	**-**	**+**
**JR-2**	**+++**	**++**	**-**	**++**
**JR-3**	**+++**	**+**	**-**	**++**
**JR-4**	**+++**	**-**	**-**	**+**
**JR-5**	**-**	**-**	**-**	**+**
**JR-6**	**++**	**-**	**-**	**++**
**JR-7**	**+++**	**+++**	**-**	**+**

+ or–: Relative activity

Selected isolate *Bacillus* sp. JR3 strain (CECT 9334) and control strain *P*. *barcinonensis* BP-23 were grown in LB for 24 hours at 30°C, under aerobic conditions. *E*. *coli* DH5α and BL21 star (DE3) strains were routinely cultured overnight at 37°C in LB broth or on LB agar plates, and were used as host strains for cloning and expression of the enzyme-encoding gene. Plasmids pGEM^®^-T Easy and pET28a/pET-101-D-TOPO^®^ (Promega/Novagen/Invitrogen^®^) were used as cloning/expression vectors producing strains *E*. *coli* DH5α/pGEMT-LipJ, *E*. *coli* BL21/pET28a-LipJ, and *E*. *coli* BL21/pET101D-LipJ-HisTag. Media were supplemented with antibiotics (ampicillin 100 μg ml^-1^; kanamycin 50 μg ml^-1^) or IPTG (isopropyl-β-D-thiogalactopyranoside; 1mM) and X-gal (5-bromo-4-chloro-3-indolyl-β-D-galactopyranoside; 80 μg ml^-1^) when required for recombinant clone selection or gene expression [28].

### DNA procedures

Genomic DNA was extracted using the GeneJet Genomic DNA Purification Kit (Thermo Scientific) according to the manufacter’s instructions. Plasmid DNA was purified using commercial kits (NucleoSpin Plasmid, Macherey-Nagel). DNA synthesis and restriction enzymes (Biotools/Thermo Scientific) were used following the manufacturer’s recommendations. PCR amplifications were performed in a Gene Amp® PCR System 2400 (Perkin Elmer) and T100™ Thermal Cycler (Bio-Rad) using different cycling periods. PCR resulting DNA was purified with Gel Band Purification kit (Thermo Scientific). To obtain the nucleotide sequences of DNA, PCR amplified fragments were analysed using the ABI PRISM^®^ BigDye^®^ Terminator v.3.1 Cycle Sequencing Kit (Applied Biosystems), and the analytical system CEQ^TM^ 8000 (Beckman-Coulter) available at the Serveis Científics i Tecnològics of the University of Barcelona. DNA samples were routinely analysed by 0.8% (w/v) agarose gel electrophoresis [28], and stained with 0.001% Nancy-520 DNA gel stain (v/v, Sigma-Aldrich). Nucleic acid concentration and purity was measured using a Spectrophotometer ND-100 NanoDrop^®^.

### Identification of strain JR3

Universal primers FW27F/BW1525R [29] were used for PCR amplification of the 16S rDNA gene from strain JR3. The complete 16S rDNA gene homology search was achieved using GenBank database. Additional information was obtained after observation of bacterial morphology and endospore location plus biochemical characterization tests including carbohydrate utilization patterns (API^®^20E and API^®^50CH systems, BioMérieux) for comparison with other strains of *Bacillus* species showing 99% identity and 100% coverage in 16S rDNA sequence (GenBank).

### Extracellular fraction preparation

Extracellular medium samples of *Bacillus* sp. JR3 were obtained from 24 h grown cultures of the strain in LB after centrifugation at 8,000 x rpm for 20 minutes. Supernatants were recovered and kept at 4°C for extracellular activity evaluation. Concentrated (50 X) supernatant samples were obtained using ultrafiltration Centricon^TM^ devices (Amicon^®^) of 10 kDa cutoff and analysed by 12% SDS-PAGE [30] after short time (0 to 5 minutes) treatments at 100°C. Zymogram analysis was performed as previously described [31,32] using 100 μM MUF-butyrate prepared in Tris HCl 20 mM buffer (pH 7) at 30 and 60°C, and visualized under UV light in a Gel Doc XR System (Biorad) before staining with Coomasie Brilliant Blue R-250 (BioRad).

### Primers design for thermophilic lipase isolation

Degenerate primers (Table 2) were designed to amplify different conserved regions found in *Bacillus sp*. and *Geobacillus sp*. thermophilic lipases, including the N- and C-terminal ends, the protein core, the active site and the oxyanion hole homolog regions. Using *Bacillus* sp. JR3 genome as a template, all possible primer combinations were used for amplification in a gradient thermocycler T100™ Thermal Cycler (Bio-Rad). 16S rDNA specific primers were used as positive control for amplification assays. Reliable PCR products were obtained with primers FWintLipGBCdeg/BWintLipGBCdeg and FWintLipBCdeg/BwintLipBCdeg, which were further sequenced and analyzed by BLASTn [33]. Those sequences showing the highest identity values were used to produce a lipase consensus sequence.

**Table 2:** Primers used for PCR amplifications (5’ to 3’ sequence).

Universal primers for bacterial 16S rDNA amplifications [29] **FW27F**: AGA GTT TGA TCM TGG CTC AG **BW1525R**: AAG GAG GTG WTC CAR CC
Degenerate primers for common regions of *Geobacillus* sp. and *Bacillus* sp. lipases **FWintLipGBCdeg**: WSNWSNAAYTGGGAYMGNGCNTGYGAR **BWintLipGBCdeg**: CCAYTGRTCNARYTTRAARTCRTA
Degenerate primers for conserved central region of *Geobacillus* sp. lipases **FWintLipGdeg**: GGNTGGGGNMGNGARGARATG **BWintLipGdeg**: NGTRTTNACDATNCCRTCRTT
Internal primers designed from the sequence resulting after alignment of the amplified fragment sequence (obtained with primers FWintLipGBCdeg and BWintLipGBCdeg) and those showing the highest similarity. NcoI and HindIII restriction sites included for cloning, highlighted.in bold **LipBFwInNcoI**: AAAAA**CCATGG**GGAAGTTATTTTTWWAAA **LipBBwInHindIII**: TTT**AAGCTT**AYTAYCTATTTCGGTAAACG
External primers designed from the sequence resulting after alignment of the amplified fragment sequence (obtained with primers FWintLipGBCdeg and BWintLipGBCdeg) and those showing the highest similarity. NcoI and HindIII restriction sites included for cloning, highlighted in bold **LipBFwOutNcoI**: AAAAA**CCATGG**AAAAATGTAAACGTTTTA **LipBBwOutHindIII**: TTT**AAGCTT**CYATCGAGAGCGCTTTATTA
Specific primers for lipJ cloning into pET101/D-TOPO® **FWLipJTOPO**: CACCATGGGGAAGTTATTTTTAAAAA **BWLipJTOPO**: TTTCGGTAATCTTAATAACTTTTCTGCAAT

### Cloning *Bacillus sp*. JR3 LipJ gene

From the consensus sequence obtained, new degenerate primers LipBFwInNcoI/LipBFwOutHindIII (Table 2) were designed and used for amplification (Expand High Fidelity polymerase, Roche) of a putative thermophilic lipase, designated LipJ, using JR3 genomic DNA as a template. The resulting PCR fragment was ligated to pGEM-T® Easy vector (Promega) and transformed into *E*. *coli* DH5α. For overexpression, pGEMT-LipJ was digested with *Nco*I/*HindI*II, ligated to the doubly digested expression vector pET28a and transformed in *E*. *coli* BL21 star (DE3). Additionally, *lipJ* gene was amplified (*Kappa*-HiFi polymerase, Kapa Biosystems) using specific primers FwLipJTOPO/BwLipJTOPO, and ligated to pET101/D-TOPO, producing recombinant plasmid pET101-LipJ-Histag, which contains the full-length enzyme linked to a C-terminal His6-tag. All constructions were verified by sequencing. The DNA sequence of *lipJ* was submitted to NCBI and given the GenBank Accession Number KU747177.

### Expression and purification of LipJ

For LipJ production, exponential growth cultures (OD_600nm_ = 0.6–0.8) of recombinant *E*. *coli* BL21/pET28a-LipJ in LB medium supplemented with kanamycin (50 μg/ml) were induced with 1 mM IPTG at different temperatures for 24 h. Cells were collected by centrifugation at 6,000 x rpm for 15 min, suspended in 20 mM Tris-HCl buffer pH 7.0 and disrupted using a SLM Aminco French Press. Clarified cell extracts were then recovered for activity determination after centrifugation at 10,000 x rpm for 10 minutes. For determination of the enzyme kinetics parameters (*Km*, *Vmax*), recombinant His6tag LipJ was purified from clarified cell extracts by immobilized metal affinity chromatography (IMAC) using HisTrap HP columns of 1 ml (GE Healthcare), and eluted in 20 mM Tris-HCl buffer (pH 7) with 500 mM NaCl using a 0 to 500 mM imidazol gradient on a fast protein liquid chromatography system (ÄKTA FPLC; GE Healthcare). Activity on *p*NP-butyrate (see below) was detected in a single elution fraction. Buffer exchange was performed by dialysis in 20 mM Tris-HCl buffer, shaking overnight at 4°C. Protein concentration was achieved in Centricon centrifugal filter units of 30-kDa molecular mass cutoff (Millipore). Bradford method was performed for protein concentration determination [34], using bovine serum albumin (BSA) as the standard.

### Activity assays

Lipolytic activity of crude cell extract, supernatant or purified samples was analysed by measuring the release of *para*-nitrophenol (*p*NP) from *p*NP-derivative fatty acid substrates (1mM for C_2_-C_5_ substrates and 0.5 mM for C_10_-C_16_ substrates, Sigma), as previously reported [35,36] using a Bio-Rad 3550 microplate reader. One unit of activity was defined as the amount of enzyme that released 1μmol of *p*NP per minute under the assay conditions used. All determinations of enzyme activity were performed by two replicas of triplicates (6 determinations per sample).

Optimum temperature of *Bacillus* sp. JR3 and *P*. *barcinonensis* supernatants was determined by analysis of the activity over a range from 4 to 100°C, at pH 7, using 1mM *p*NP-butyrate as a substrate [35]. Thermal stability was determined by incubating cell extracts or supernatant samples at temperatures from 4 to 100°C for 1 to 96 h; residual activity was measured under standard assay conditions once samples reached the optimum temperature. pH stability of samples was determined by measuring the residual activity on *p*NP-butyrate after 1 h incubation at different pH. The effect of temperature or pH on LipJ lipolytic activity was evaluated by response surface methodology (RSM) [37], as stated below. To evaluate the effect of metal ions or inhibitors on activity, assays were performed on *p*NP-butyrate in the presence of several metal chlorides (Al^+3^, Ba^+2^, Ca^+2^, Cu^+2^, Fe^+2^, Hg^+2^, K^+^, Li^+2^, Mg^+2^, Mn^+2^, Na^+^, NH_4_^+^, Zn^+2^) and EDTA, used at different concentrations (1 and 10 mM). Moreover, Ca^+2^ (1–30 mM) and Zn^2+^ (0–5 mM) were assayed at 30°C and 60°C and in the range 60–100°C. Residual activity was measured at 30°C and pH 7 using a conventional assay on *p*NP-butyrate, and expressed as a percentage of activity without ions or inhibitors, respectively. Supernatant samples of *Bacillus* sp. JR3 were also incubated in the presence of 1 mM PMSF (phenylmethylsulfonyl fluoride, Sigma) to measure the effect of this serine inhibitor on enzyme activity. Kinetic parameters (*Vmax* and *Km*) were determined under optimal assay conditions by fitting hyperbolic Michaelis-Menten curves with GraphPad Prism ® software version 6.

### Statistical analysis

For optimum pH and temperature determination, response surface methodology (RSM) [37] was applied, using a design of experiment of 22 Central Composite Designs (CCD) with 5 levels leading to 11 sets of experiments with 3 replicates of the central position. Temperatures ranging from 16 to 50°C and the Britton-Robinson 50 mM buffer adjusted to pH ranging from 4.2 to 9.8 were used. The experimental results of CCD, represented by the mean of at least 6 samples each, were fitted to the following second order polynomial equation: (*z* = −186.5841+48.6774*x*−3.4315*x*^2^+2.9364*y*−0.0500*y*^2^−0.0173*xy*). Quality of this equation was evaluated by the coefficient of determination R-*sqr*, which was 0.94557, indicating that 94.56% response data can be justified by the chosen model. Table 3 shows that pH and temperature (°C), and the interaction of both of them (1L by 2L), do not fit a linear regression, as P > 0.005, and both variables are independent. However, these variables can be adjusted to a quadratic model regression with a 90% confidence level, as P < 0.05. Therefore, the model used allowed prediction of any activity value, providing values of pH and temperature with a precision of 94.56% and 90% confidence. The statistical significance of the Central Composite Design model was determined by the *F*-test ANOVA. Statistical analysis and RSM values were calculated with STATISTICA software version 8.

**Table 3:** Statistical analysis.

ANOVA; Var.:Act (U mL^-1^); R-sqr = 0.94557; Adj:0.89114 (Spreadsheet 3) 2 factors, 1 block, 11 Runs; Mean Square Pure Error = 0.8567327 DV: Act (U mL^-1^)					
	Addition of Squares	df	Mean of Squares	F	Probability
**(1)pH (L)**	0.01655	1	0.01655	0.01931	0.902203
**pH (Q)**	49.47081	1	49.47081	57.74358	0.016881*
**(2)Temp (°C)(L)**	2.03925	1	2.03925	2.38026	0.262839
**Temp (°C)(Q)**	29.32510	1	29.32510	34.22900	0.027994*
**1L by 2L**	0.03454	1	0.03454	0.04031	0.859435
**Lack of Fit**	1.96037	3	0.65346	0.76273	0.610212
**Pure Error**	1.71347	2	0.85673		
**Total SS**	67.49368	10			

*Statistically significant at 90% probability level

### Bioinformatics tools

BLAST searches were routinely performed for DNA or protein sequence analysis and to retrieve identity and similarity percentages by pairwise alignment [33]. Multiple sequence alignments were obtained using T-Coffee (http://tcoffee.crg.cat/) [38] or ClustalO (http://www.ebi.ac.uk/Tools/msa/clustalo/) [39]. BioEdit v.7.0.1 [40] was used for restriction and consensus pattern determination. Identification of putative signal peptide [41] was performed through SignalP versions 3.0 or 4.0 (http://www.cbs.dtu.dk/services/SignalP/), and transmembrane regions determined through TMHMM tools (http://www.cbs.dtu.dk/services/TMHMM-2.0). ExPASy proteomics server was used to analyse the protein physico-chemical parameters (ProtParam tool; http://web.expasy.org/protparam/), and to predict isoelectric point and molecular mass of the cloned protein [42]. Detection of aromatic amino acids was performed using GPMAW tool (http://www.alphalyse.com/customer-support/gpmaw-lite-bioinformatics-tool/start-gpmaw-lite/) [42]. ProDom (http://prodom.prabi.fr/prodom/), Pfam (http://pfam.xfam.org/), and InterProScan 5.2 (http://www.ebi.ac.uk/interpro/) were used for domain identification [43], and a Hidden Markov Model was created with HMMER (http://www.ebi.ac.uk/Tools/hmmer/) in order to detect conserved protein motifs [44]. LipJ amino acid sequence was compared with PDB database to identify possible template proteins with available 3D structure. *Pelosinus fermentans* lipase PfL1 (pdb *5AH0*, 92% coverage and 56% identity) [45] and *Geobacillus stearothermophilus* lipase L1 (pdb *1KU0*, 92% coverage and 49% identity) [46] were selected for homology model construction and validation using Phyre2 server (http://www.sbg.bio.ic.ac.uk/~phyre2/html/page.cgi?id=index) [47]. 3D structure superposition and search for conserved amino acid motifs or ion binding cavities was performed using the visualization tool Pymol Molecular Graphics System, Version 1.5.0.4, Schrödinger, LLC (http://www.pymol.org). For family assignment, a phylogenetic analysis was conducted using MEGA 7 software including lipase sequences of functionally characterized enzymes bearing similar catalytic mechanisms, available in the literature and in UniprotKB (http://www.uniprot.org/) or in Protein Data Bank (http://www.rcsb.org/pdb/) databases, always considering their catalytic and conserved residues [48,49]. A phylogenetic tree was constructed applying the Maximum Likelihood (PALM) method, employing WAG+G as the amino acid substitution model [50]. A bootstrap consensus tree was achieved after 1000 repeats, with a cut off of 50% [50]. Family assignment was confirmed using ESTHER database (http://bioweb.ensam.inra.fr/esther), specific for α/β-hydrolase fold protein superfamily [51].

## Results and discussion

### *Bacillus* sp. JR3 isolation

A green laurel forest located at the volcanic island of El Hierro (Canary Islands, Spain) was chosen as a source for isolation of spore-forming bacteria coding for exceptional enzymatic activities resulting from natural evolution of volcanic soils, and due to their capacity to persist in extreme environments. Seven Gram-positive bacterial sporulated isolates (JR1 to JR7) showing either protease, cellulase, lipase or esterase activity were obtained after growth at mild temperature (30°C) for 24 h in aerobic conditions (Table 1). Among them, strain JR3 was selected for further identification and characterization due to its differential morphology and strikingly high esterase activity when culture supernatants were assayed on *p*NP-butyrate at 60°C. BLAST analysis of the 16S rDNA sequence of strain JR3 allowed assigning this isolate to the genus *Bacillus*, in close proximity to the *B*. *cereus* and *B*. *thuringiensis* group (query cover 100%, maximum identity 99%). Additional API^®^20E i API^®^50CH tests were performed and the results obtained again assigned the strain to the genus *Bacillus*, almost identical to *B*. *cereus*, although no complete match was obtained for gelatine utilization and acetoin production, which resulted positive and negative, respectively. Therefore, the strain was named *Bacillus* sp. JR3.

### Extracellular esterase activity of *Bacillus* sp. JR3

**Fig 1:**
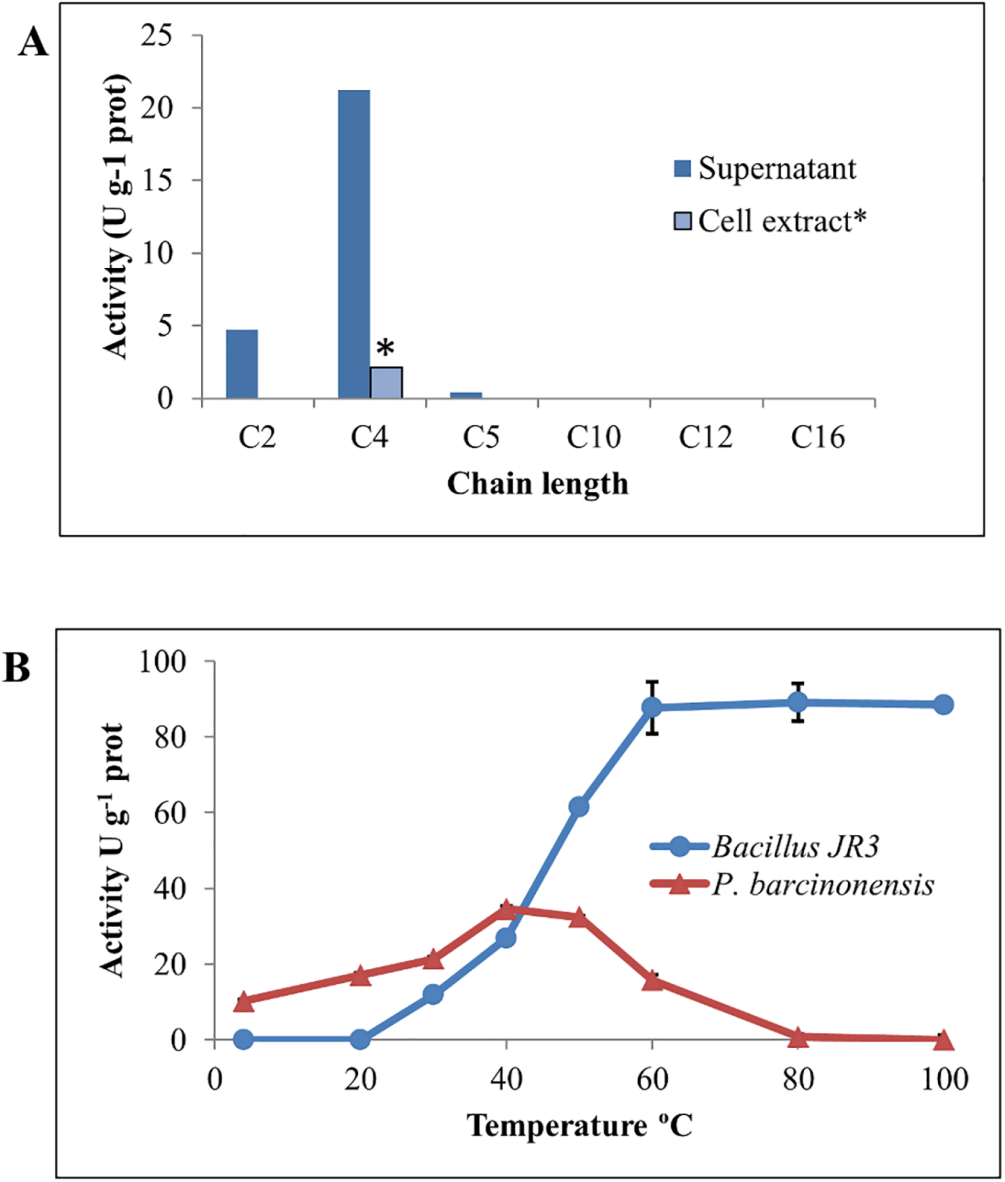
Activity assays of *Bacillus* JR3 supernatants. (A) Substrate specificity profile; *cell extract activity was only assayed on *p*-NP-butyrate. (B) Activity at different temperatures of strain JR3 (blue diamonds) and *P*. *barcinonensis* (red squares), the latter used for comparison of mesophilic activity.

Extracellular lipase activity of strain JR3 was initially tested on different chain-length *p*NP-derivative substrates at 37°C. Supernatants of the strain grown at 30°C for 24 h showed activity on C_2_ to C_7_ substrates (Fig 1A). However, under the conditions used here (15 minute incubation assays), no significant activity could be detected on substrates of longer chain length (C_10_ to C_18_). Maximum activity at 37°C was obtained on *p*NP-butyrate (21.23 U g^-1^), whereas activity on *p*NP-acetate and *p*NP-valerate was 4.72 and 0.43 U g^-1^, respectively. Activity assays performed using crude cell extracts of the strain grown under the same conditions revealed also the presence of a faint intracellular activity, with a maximum value at 37°C of 2.11 U g^-1^ on *p*NP-butyrate (Fig 1A*).

The apparent kinetic parameters of supernatant samples from *Bacillus* sp JR3 were determined at 37°C using *p*NP-butyrate. A typical Michaelis-Menten kinetics plot was obtained (not shown), with a *Km* of 0.076 mM and a *Vmax* of 0.522 U mg^-1^, showing lack of interfacial activation, therefore suggesting that the major extracellular lipolytic activity of the strain corresponds to esterase, according to the proposed classification based on substrate specificity [2].

**Fig 2:**
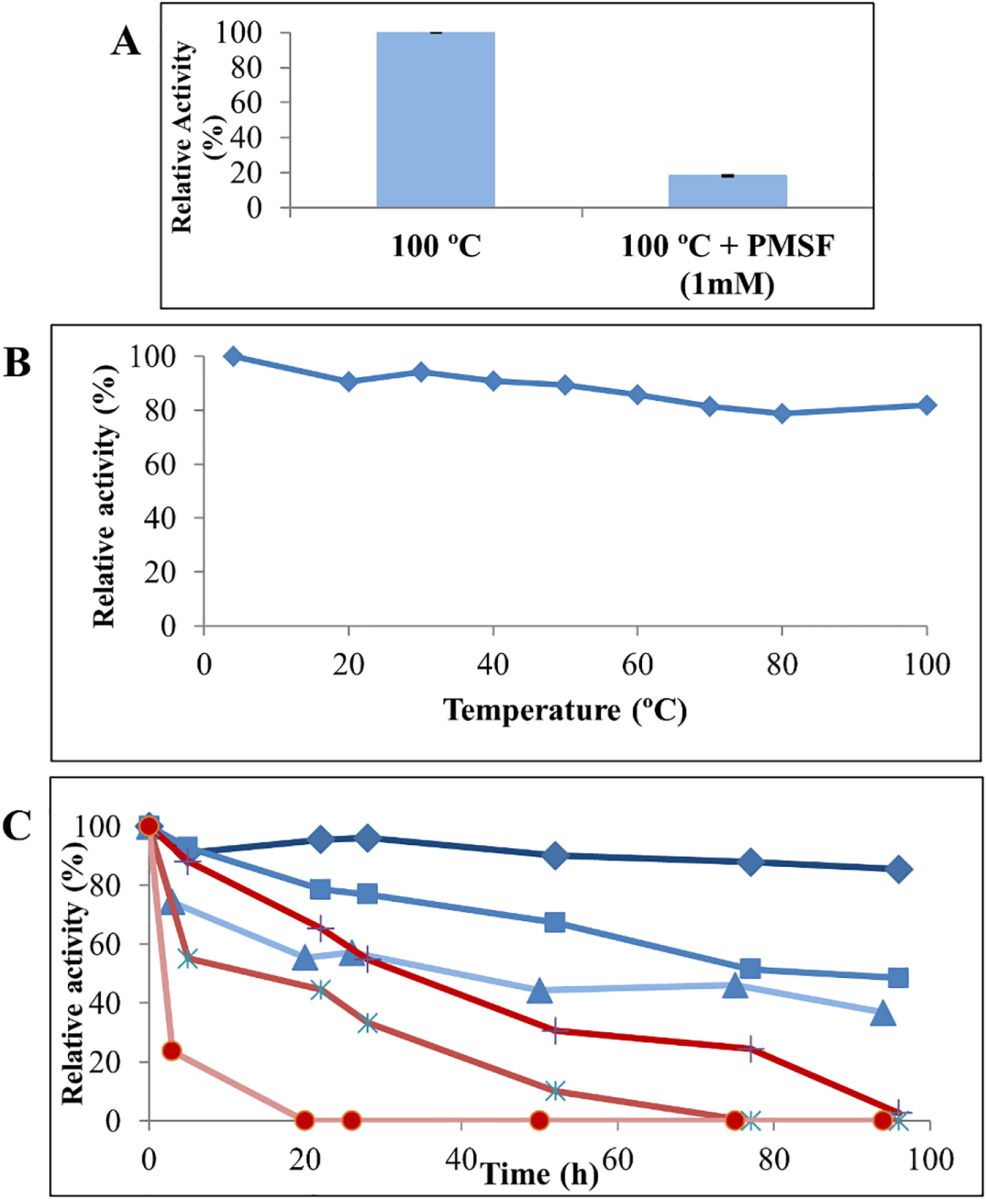
Temperature assays of *Bacillus* JR3 extracellular activity. (**A**) Activity assay at 100°C with and without PMSF. (**B**) Residual activity found after 15 min incubation in a temperature range from 4 to 100°C. (**C**) Long-term thermal stability of JR3 (blue diamonds, blue squares, blue triangles) and *P*. *barcinonensis* (black +, blue Ӿ, red dots) supernatants, incubated for 90h at 60 (blue diamonds, black +), 80 (blue squares, blue Ӿ) and 100°C (blue triangles, red dots). *P*. *barcinonensis* supernatant activity measured for comparison of mesophilic activity.

Optimum temperature of strain JR3 supernatants was determined as described in Materials and Methods, resulting in a strikingly high activity at temperatures over 60°C (Fig 1B), reaching maximum activity at 80–100°C (ca. 90 U g^-1^). To assess that the results obtained were not due to an artefact caused by the effect of such high temperatures on substrate or buffer stability, supernatants from *P*. *barcinonensis* BP-23 [27] were used as internal control and assayed for activity over the same range of temperatures. *P*. *barcinonensis* displayed a typical mesophilic lipase activity profile compared with that of supernatants from *Bacillus* sp. JR3 (Fig 1B), suggesting the existence of a thermophilic lipolytic extracellular system in JR3. To further confirm this interesting behaviour, new activity assays were performed at high temperatures in the presence of PMSF. PMSF is a common serine inhibitor of enzymes bearing a nucleophilic serine at the active site [15], as is the case for lipases [2,10]. Assays performed for only 15 minutes at 80°C showed almost complete inhibition (81% activity loss) of activity in supernatant samples of strain JR3 in the presence of PMSF, whereas full activity was achieved when PMSF was not present in the reaction medium (Fig 2A). These results unambiguously confirm that the activity found at high temperatures in supernatants of strain JR3 was not artefactual but it was indeed due to the presence of at least a thermophilic lipolytic enzyme. However, it must be stated that the strain was isolated at 30°C and could not grow when incubated at temperatures over 50°C, indicating that it is in fact a mesophilic strain, yet bearing thermophilic esterase activity, probably as a reminiscence of its life under the ancient extreme environmental conditions of the volcanic soil.

Thermal stability was assayed by measuring the residual activity after incubation of supernatant samples of strain JR3 in a temperature range from 4 to 100°C for 15 minutes. Almost complete activity recovery was obtained at all assayed temperatures (Fig 2B). Moreover, thermal stability assays performed at 60, 80 and 100°C for 96 hours showed a 50% activity loss only after 50 and 96 hours incubation at 100 and 80°C, respectively, while supernatants of *P*. *barcinonensis* rapidly lost their activity at high temperatures (Fig 2C). Interestingly, only a 15% loss of activity was observed after 96 hours incubation at 60°C, thus confirming the interesting thermoresistance and thermophilicity of JR3 extracellular lipolytic activity (Fig 2C), which could meet the conditions of biotechnological processes requiring high temperatures and long incubation times [52]. Therefore, we focussed on the prospection of thermophilic lipases/esterases in the genome of strain JR-3.

### Gene isolation and cloning

**Fig 3:**
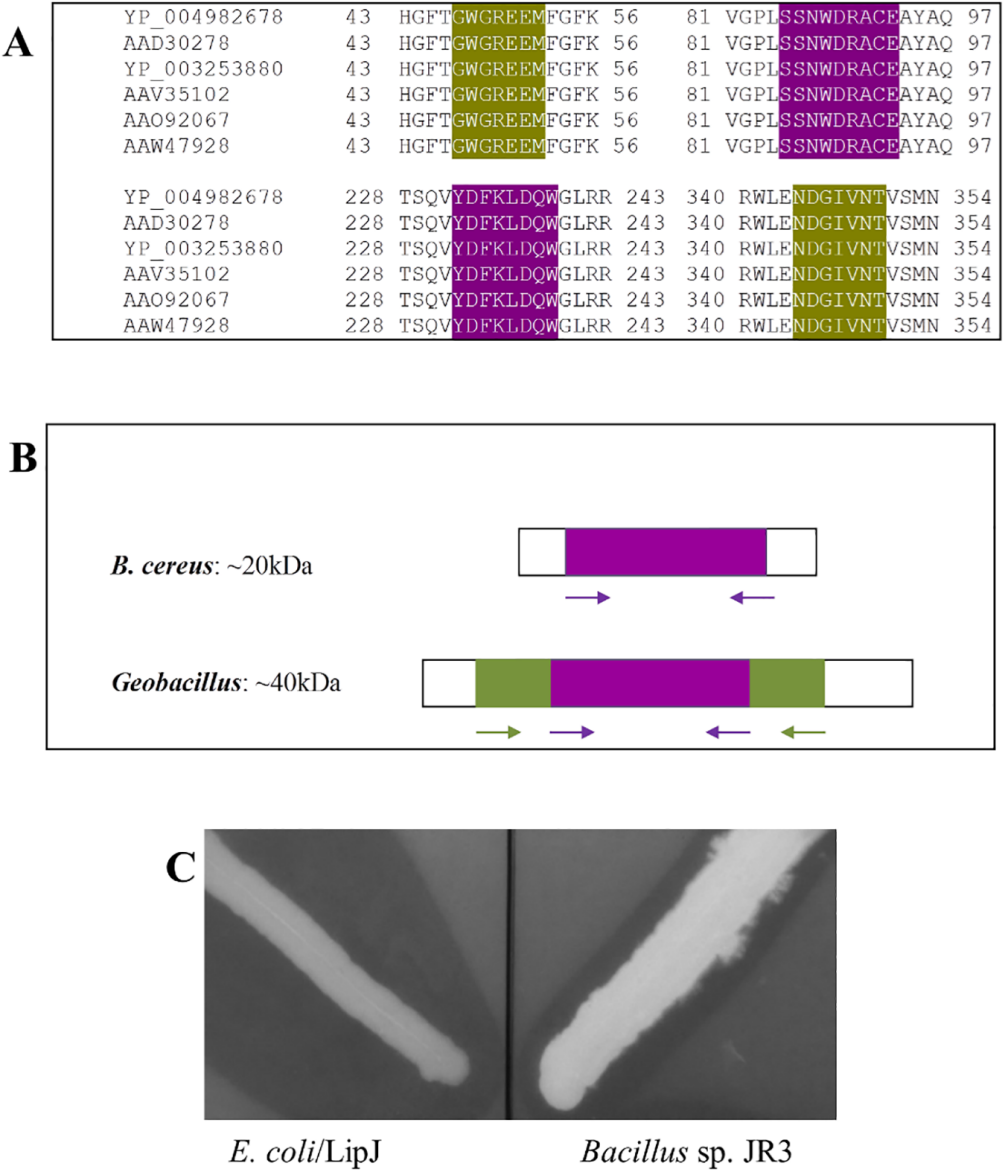
Conserved motifs (**A**) and regions (**B**) of short *Bacillus* and long *Geobacillus* lipases found in the databases, used for the design of consensus degenerated primers employed for amplification of an internal lipase coding DNA fragment from strain JR3. (C) Tributyrin-supplemented plate assay showing the hydrolysis haloes produced by cloned LipJ (left) and strain JR3 (right).

Search for thermophilic *Bacillus* sp. or *Geobacillus* sp. lipase sequences in the databases revealed that they all belong to the bacterial lipase family I.5 [10]. Among the lipases from these genera, two clusters were established upon multiple sequence alignment (Fig 3A and 3B): those belonging to the *B*. *cereus* group (20–35 kDa), and those of *Geobacillus* group (40–45 kDa), a genus that was created to include thermophilic members of former *Bacillus* species [53]. Both clusters share a common central region of ca. 480 bp (Fig 3A and 3B) for which the set of degenerate primers, FWintLipGBCdeg/ BWintLipGBCdeg (conserved motifs SSNWDRACE and YDFKLDQW) was designed (Table 2). On the other hand, lipases from the *Geobacillus* cluster are longer in size, and contain two conserved sequence fragments flanking the central common region. A new set of degenerate primers, FwintLipGdeg/ BWintLipGdeg (conserved motifs GWGREEM and NDGIVNT) was designed (Table 2) for amplification of a putative thermophilic *Geobacillus*-like lipase sequence. Both sets of primers produced amplicons of ca. 450 and 900 bp, respectively, which were aligned with the closest sequences in the databases to produce a consensus sequence that was used to design two new sets of degenerate primers (Table 2), one inside (LipBFwInNcoI, LipBBwInHindIII) and another outside (LipBFwOutNcoI, LipBBwOutHindIII) the consensus sequence coding region. The four primer combinations were tested for amplification using *Bacillus* sp. JR3 genomic DNA as a template, and a band of ca. 1300 bp was obtained with primers LipBFwInNcoI and LipBBwOutHindIII (Table 2). The amplified 1300 bp DNA fragment was sequenced and analysed by BLASTn, showing 91–96% identity and 92% coverage with predicted lipase sequences belonging to the *B*. *cereus* group. This fragment, designated *lipJ*, was further digested with *Nco*I and *Hind*III, and cloned in *E*. *coli* BL21 using vectors pGEM-T^®^, TOPO and pET28. Presence of *lipJ* insert in the resulting recombinant clones was confirmed by restriction digestion and sequencing, showing the presence of an ORF of 1242 bp coding for a hypothetical protein of 413 amino acids, with a predicted molecular mass of 46 kDa and a *p*I of 6.5, displaying activity on tributyrin (Fig 3C). The complete sequence of *lipJ* was submitted to GeneBank and given the accession number KU747177.

### LipJ purification and characterization

**Fig 4:**
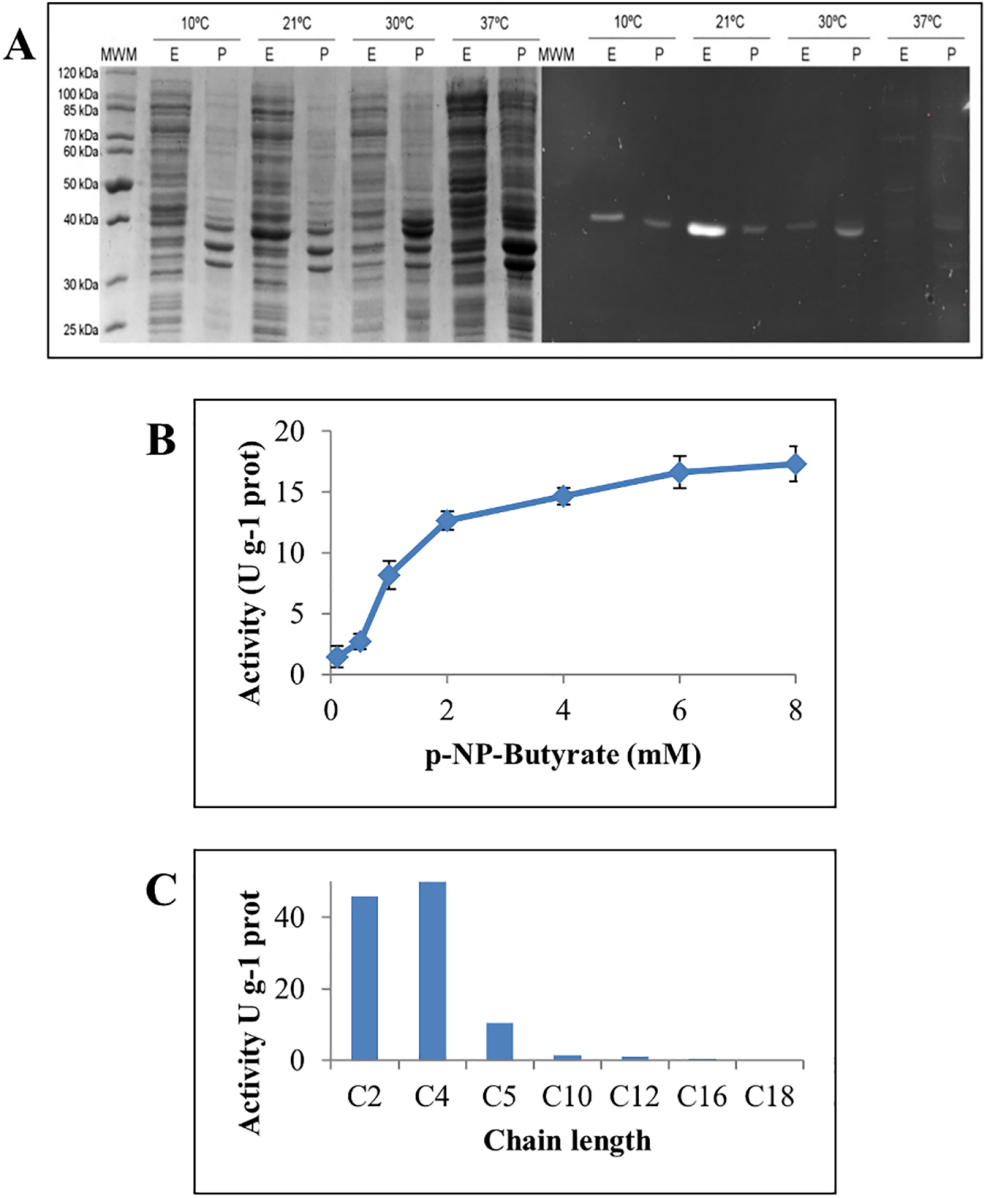
Expression and activity profile of cloned LipJ. (**A**) Coomassie-stained (left) and zymogram [31,32] (right) SDS-PAGE of crude cell extracts (E) and cell debris (P) of cloned LipJ induced with 1 mM IPTG at different temperatures. (**B**) Michaelis-Menten kinetics profile of cloned LipJ. (C) Substrate specificity of cloned LipJ assayed on *p*NP-derivative substrates of different chain length.

Expression assays of *E*. *coli* BL21/pET28-LipJ were done after IPTG induction at different temperatures (10°C, 21°C, 30°C and 37°C). Activity of soluble and insoluble fractions of induced cell extracts was analysed by zymogram [31,32], showing a band of ca. 40 kDa with activity on MUF-butyrate at 30°C (Fig 4A). The highest expression was obtained in soluble fractions of cell extracts induced at 21°C with 1 mM IPTG.

For characterization purposes, LipJ was purified by fast protein liquid chromatography from 20-fold concentrated crude cell extracts of recombinant *E*. *coli* BL21/pET101D-LipJ-HisTag. The purification process rendered a low yield (5.3%) that allowed isolation of a small sample of semi-purified protein. Unexpectedly, the resulting semi-purified enzyme did not display activity at 80°C, showing also very low activity at 37°C, thus allowing only the study of the kinetic parameters of LipJ. When assayed at 37°C on *p*NP-butyrate, the enzyme displayed a typical Michaelis-Menten plot (Fig 4B) without interfacial activation, like most esterases, with a calculated apparent *Km* and *Vmax* of 1.7 mM and 21.6 U g^-1^, respectively. Further characterization of LipJ was therefore performed using crude cell extracts of *E*. *coli* BL21/pET28-LipJ, prepared as described in Materials and Methods.

LipJ substrate specificity was assayed at 37°C on several *p*NP-derivatives, showing preference for short chain-length fatty acid substrates (Fig 4C), and exhibiting the highest activity (52.1 U g^-1^; 100%) on *p*NP-butyrate (C_4:0_). The enzyme maintained almost 95% activity on *p*NP-acetate (C_2:0_; 45.7 U g^-1^) and 20% on *p*NP-valerate (C_5:0_; 10.3 U g^-1^). However, a dramatic activity reduction was observed when long chain length substrates were used under the same conditions (Fig 4C).

**Fig 5:**
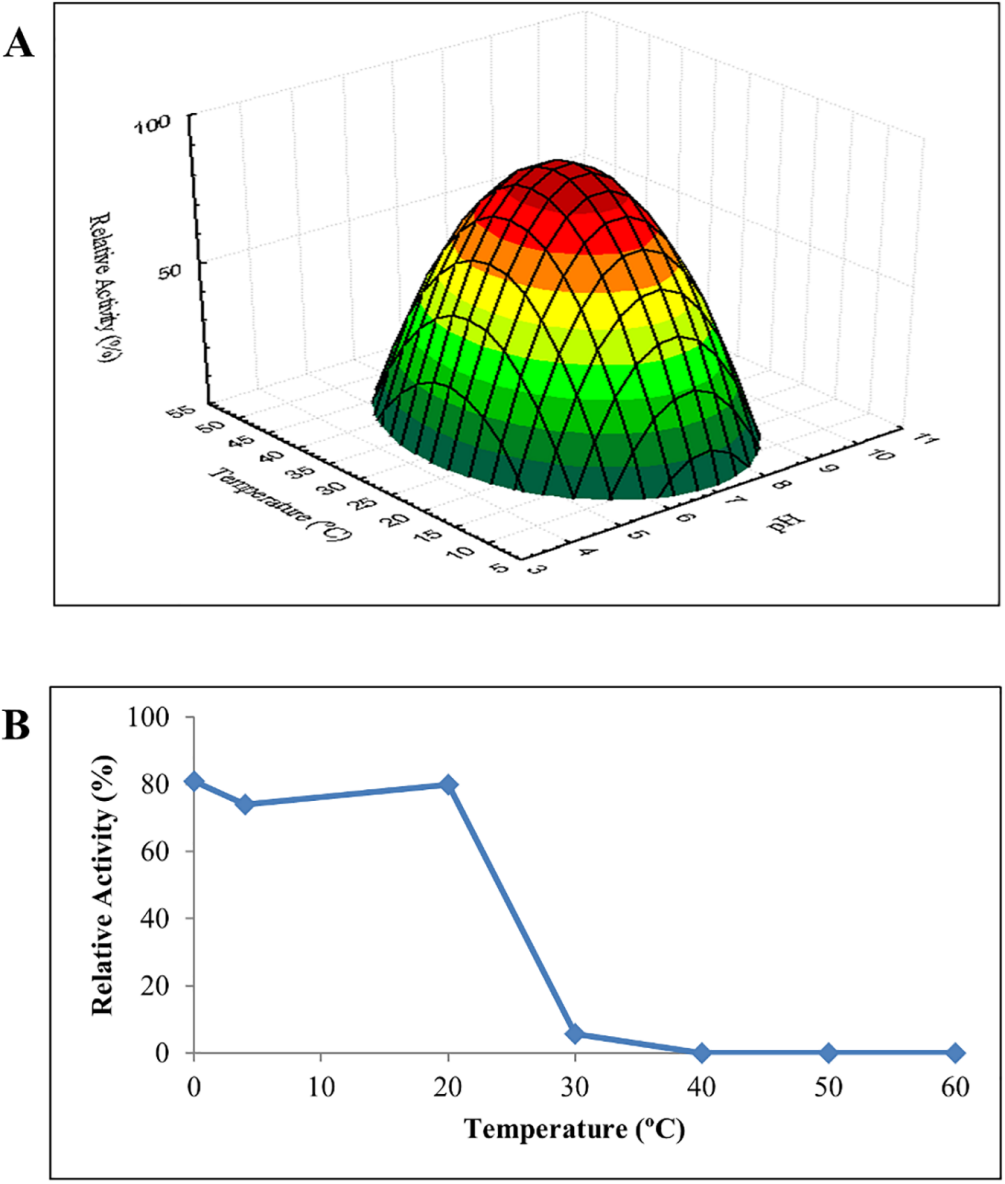
Properties of cloned LipJ. (**A**) RSM results plot for optimum temperature and pH determination. (**B**) Thermal stability of LipJ cell extracts incubated for 1h at different temperatures.

The effect of temperature and pH on the activity of LipJ was determined on *p*NP-butyrate (Fig 5A), using a *Surface Response Methodology* strategy [37]. Surprisingly, LipJ displayed maximum activity at pH 7.0 and 28.13°C, whereas no activity could be detected at extreme pH or high temperatures. Moreover, thermal stability assays performed at optimum pH and temperature on *p*NP-butyrate demonstrated that the cloned enzyme was rapidly inactivated when incubated at temperatures over 30°C (Fig 5B). These results are in clear contradiction with the extracellular thermophilic profile found in the supernatant of strain *Bacillus* sp. JR3, and suggest that the cloned enzyme LipJ is not the thermophilic enzyme we intended to clone. On the contrary, the catalytic behaviour of LipJ points to a mesophilic esterase that could even be intracellular, based on the fact that most secreted *Bacillus*-related lipases are alkaliphilic [12,54,55], while LipJ only shows activity at neutral pH, as happens for most intracellular esterases [56–58].

Thus, taking into consideration the previous results and on view of the kinetic parameters displayed by LipJ, with lack of interfacial activation, we propose that LipJ should be considered an esterase showing similar properties to those described for other *Bacillus*-related species carboxylesterases like *B*. *subtilis* PnbA [59], *P*. *barcinonensis* BP-23 EstA [56], or *Bacillus* sp. BP-7 EstA1 [57]. Nevertheless, these results are also in contradiction with those expected from the PCR prospection procedure because gene *lipJ* displays similar length and high sequence identity with thermophilic lipases of both, *Bacillus* and *Geobacillus* genera, and the gene was isolated from a strain (JR3) bearing proven thermophilic lipolytic activity. To find an explanation for the results obtained, zymogram analysis [31,32] and a bioinformatics approach were addressed.

### Zymogram analysis

**Fig 6:**
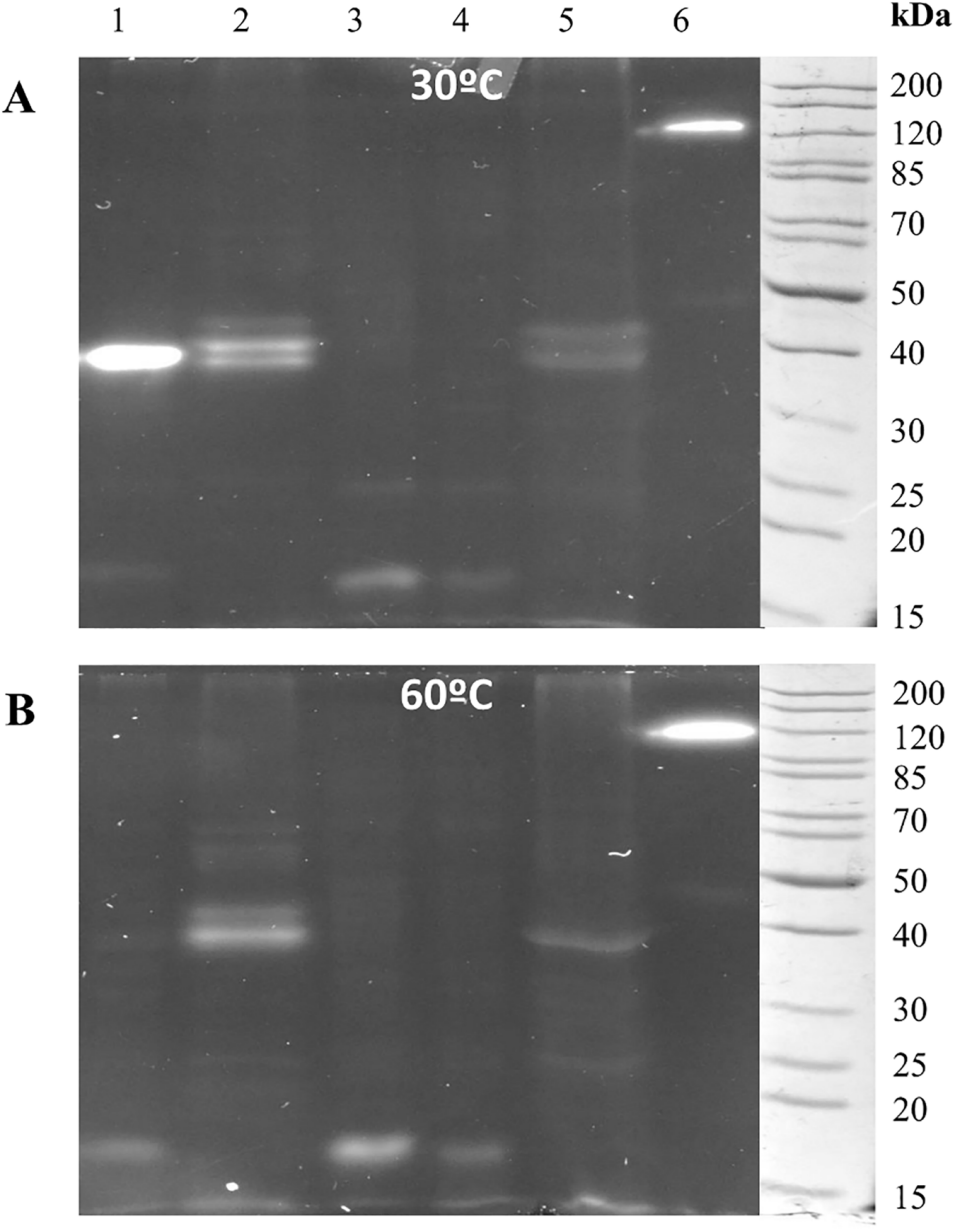
Zymogram analysis [31,32] of cell extract and supernatant samples of cloned LipJ and *Bacillus* JR3, performed at 30°C (**A**) and 60°C (**B**). Lane 1: crude cell extract of *E*.*coli* expressing LipJ; Lane 2: supernatant of *B*. *cereus* 131, used for comparison; Lanes 3–4: soluble (3) and insoluble (4) cell extract of *E*.*coli* without the *lipJ* insert, used as a negative control; Lanes 5–6: concentrated supernatant (5) and crude cell extract (6) of *Bacillus* strain JR3.

Zymogram analysis [31,32] of cloned LipJ and concentrated samples of *Bacillus* sp. JR3 supernatant and cell extracts was performed at 30°C and 60°C on MUF-butyrate (Fig 6). For technical reasons, no higher temperatures were tested. As internal controls, soluble and insoluble fractions of cell extracts from *E*.*coli* bearing the same plasmid but without the *lipJ* insert were analysed (Fig 6, lanes 3 and 4), together with concentrated supernatant samples of *B*. *cereus* 131 (Fig 6, lane 2). As shown in Fig 6A, cloned LipJ appears as a band of ca. 40 kDa, whereas supernatants of *Bacillus* sp. JR3 display a complex lipolytic system including a set of bands of ca. 44, 39, and a fainter band of 22 kDa when assayed at 30°C (Fig 6A). Non boiled cell extracts of strain JR3 show a faint 49 kDa band plus a high molecular weight activity band of ca. 120 kDa probably due to aggregates, a very common trait among lipases [58,60]. It is interesting to note the differences in the activity band pattern of *B*. *cereus* (CECT131) and that of strain JR3, showing similar but not identical bands, in agreement with the above data indicating that both strains are close to each other but not identical. When the same samples were analysed in zymograms performed at 60°C [31,32], the band corresponding to LipJ disappeared, indicating loss of activity of the cloned enzyme at this temperature (Fig 6B). On the contrary, both supernatant and cell extract samples of strain JR3 show bands with activity at this temperature. The extracellular 39 kDa band almost disappears but the 44 kDa band still displays high activity at 60°C, confirming the presence of at least an extracellular thermophilic lipase (Fig 6B). Also the 120 kDa and the faint 49 kDa band found in cell extracts of JR3 show higher intensity when assayed at 60°C. The apparent high molecular mass of these bands suggest that they might correspond to the aggregated and unprocessed form of the extracellular thermophilic lipase. The results obtained here confirm the mesophilic profile of LipJ and its differential behaviour in comparison to the extracellular thermophilic lipase activity of strain JR3 and are in agreement with the previous observation of inactivation of LipJ at temperatures over 30°C (Fig 5B). Additionally, the 22 kDa band could be the common low molecular mass lipase of most *Bacillus*-related species, a well-known ubiquous lipase previously reported [61,62], which has seldom been described as a thermotolerant enzyme [63,64]. On the other hand, considering its size and activity at 60°C, the 44 kDa band could be assigned to the thermophilic activity found in supernatants of the strain JR3, being different from cloned LipJ. Although this band was isolated and analysed by MALDI-TOF, no alpha/beta-hydrolase (neither lipase nor esterase) domains could be identified, except for a fragment corresponding indeed to a section of LipJ sequence.

### LipJ sequence and structure analysis

As stated above, the nucleotide sequence of gene *lipJ* showed a single open reading frame of 1242 bp coding for a hypothetical protein of 413 amino acids, with a predicted molecular mass of 46.2 kDa and a *p*I of 6.46. A putative Shine-Dalgarno (AGTGA) sequence was found 9 nucleotides upstream the initiation codon [40], along with a -10 (AATTCA) putative promoter region. Three TAA contiguous repeats were found downstream the stop codon but neither inverted repeats nor significant secondary structures that could act as transcription terminators appeared at the available intergenic region, suggesting that other signals located downstream could serve for transcription termination [40].

Being strain *Bacillus* sp. JR3 closely related to *B*. *cereus* and *B*. *thuringiensis* group, a BLASTp search for LipJ-homologous proteins in these genera (*B*. *cereus*, tax id. 1396 and *B*. *thuringiensis*, tax id. 1428) was performed. An annotated lipase (100% coverage and 97% identity) and a hypothetical protein (100% coverage and 93% identity) were found, respectively, both uncharacterized so far. This positions LipJ as the first functionally characterized enzyme with this specific amino acid sequence in the *B*. *cereus* group.

A typical carboxylesterase consensus pentapeptide–GX**S**XG–was found in the deduced amino acid sequence of the cloned enzyme, including Ser^138^ as a part of the predicted catalytic triad, together with residues Asp^340^ and His^380^, assigned by similarity [45,46,65]. Interestingly, an additional pentapeptide-like motif (AA**S**^214^FG) bearing an alanine at the first position, like in most secreted *Bacillus* lipases, was detected. However, further structure analysis of LipJ (see below) positioned this motif far from a putative active site, suggesting that it is not indeed a functional lipase pentapeptide. Although being an infrequent trait among lipases, presence of more than one pentapeptide-like motifs has already been described for *Pseudomonas* CR611 Lip I.3 acidic lipase [66] and is also found in many *Bacillus/Geobacillus*-related thermophilic lipase sequences from the databases (UniProt). Also, a bacterial oxyanion hole-like motif (P^35^IILVNG) was identified [67], which matched well with those of *Geobacillus* thermophilic lipases [68].

Knowing that most *Bacillus*-*Geobacillus* lipases are secreted extracellularly, a search for a signal peptide was performed on the amino acid sequence of LipJ. Curiously, depending on the SignalP program version used [69], different results were obtained: when SignalP 4.1 was used with default settings, no apparent signal peptide existed in LipJ; however, using the default cut-off values of SignalP 3.0 for Gram-positive bacteria, a signal peptide was identified with a probability of 0.994 and the processing point located between amino acids 28 and 29 (AEE-K) [41,70]. This observation could explain the difference in size of recombinant LipJ expressed in *E*. *coli* when assayed in zymograms (Fig 6) and the predicted molecular weight.

To get a better knowledge of LipJ properties, we analysed and compared the sequences of related enzymes, extracted from the databases. According to protein domain databases, LipJ is a single domain, globular protein, containing the signature of α/β-fold hydrolases with the consensus pattern _PS00120, unambiguously identified as a lipolytic enzyme [8,71]. This assignment is supported by the high content of non-polar amino acids (hydrophobicity index -0.34) found in the protein sequence [72]. Secondary structure prediction confirmed the typical α/β-fold of carboxylesterases and location of the conserved pentapeptide constituting the “nucleophilic elbow” between strand β3 and the following α helix [2,72].

**Fig 7:**
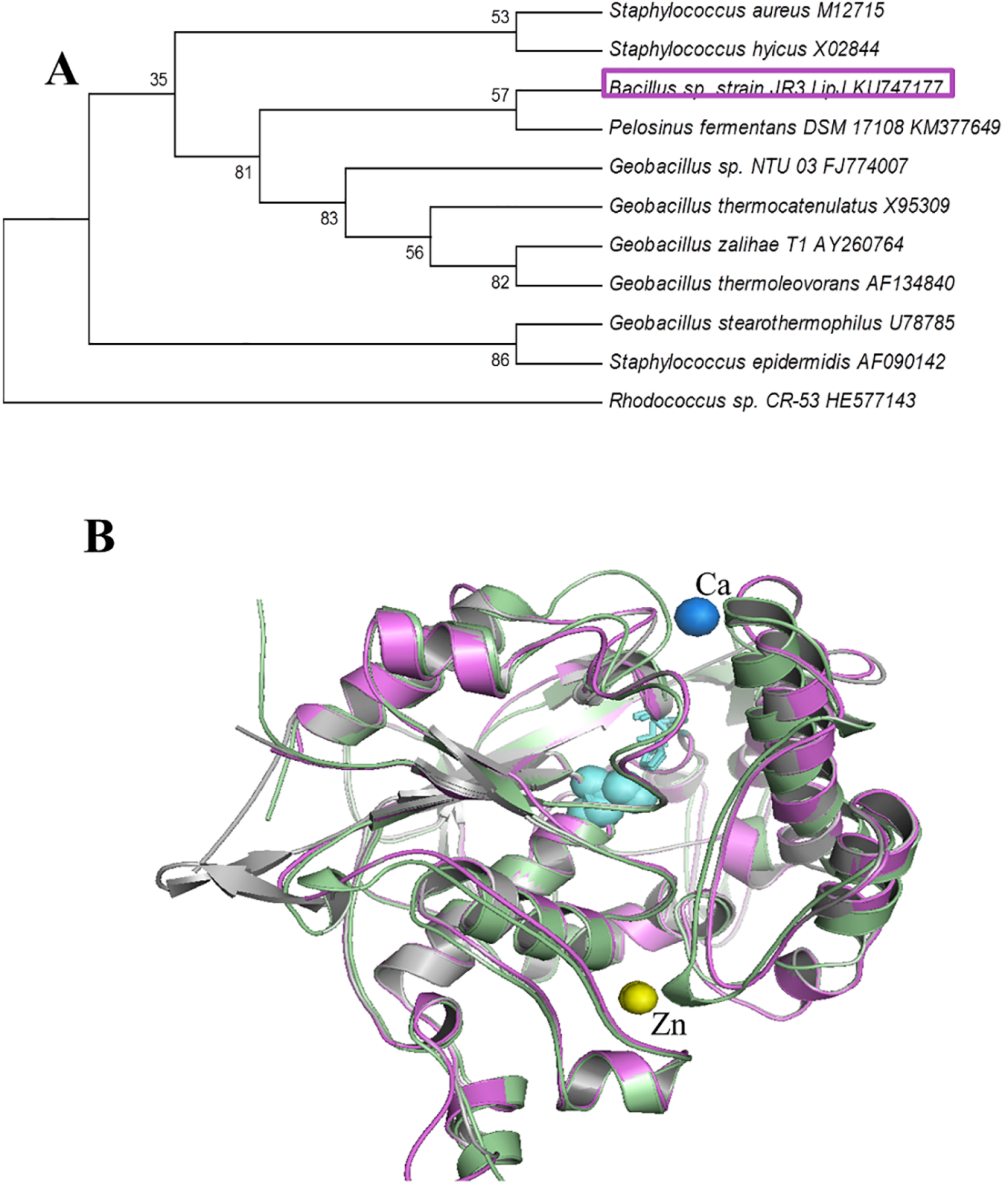
LipJ *in silico* analysis. (**A**) Phylogenetic tree of LipJ obtained using MEGA 7 software [50] and the described family I.5 lipases plus the closest *Pelosinus fermentans* lipase PfL1. The box highlights the position of LipJ, grouping in the same cluster as family I.5 thermophilic lipases. A *Rhodococcus sp*. *CR53* family X lipase [11] was included in the tree for external rooting. (**B**) 3D model structure obtained for LipJ (purple), aligned and superposed with those of *G*. *stearothermophilus* L1 (green) and *Pelosinus fermentans* PfL1 (grey). The three structures show almost a complete match except for two external β-sheets emerging from the structure of *P*. *fermentans* PfL1 (left). The residues involved in catalysis appear at the same position in the three structures and have been highlighted in cyan. Zn and Ca ions (yellow and blue spheres) show the position of the putative binding cavities, which might also be present in LipJ.

Assignment of LipJ to a defined family or cluster was performed by inspecting the sequence/function similarities [10], and construction of a phylogenetic tree. LipJ displays significant similarities to the motifs that define the bacterial lipase family I.5, which includes thermophilic lipases from *Bacillus*, *Geobacillus* and *Staphylococcus* species [9,10], suggesting that LipJ might belong to this family. In fact, a phylogenetic tree constructed using the described family I.5 lipases [10,73], positioned LipJ in the same cluster, close to *Bacillus/Geobacillus* lipases (Fig 7A). Moreover, the database ESTHER, specific for α/β-hydrolases, also assigned LipJ to the *Bacan*-BA2607 group (Bacterial_lip_FamI.5, including *B*. *anthracis*, *B*. *thuringiensis* and *B*. *cereus*) [51], giving support to this assumption.

Two validated 3D homology models of LipJ were constructed using pdb *5AH0*, a family I.5 lipase from the anaerobic groundwater organism *Pelosinus fermentans* showing 56.3% sequence identity and 90% coverage [45], and pdb *1KU0*, corresponding to L1, a thermoalkaliphilic secreted family I.5 lipase from *Geobacillus stearothermophilus* [65]. In both cases the 3D models were identical, being validated with 100% residues modelled at >90% confidence. Position of the catalytic triad Ser^138^, Asp^340^ and His^380^, and the putative oxyanion hole (P^35^IILVHG) [68,74,75] at the expected positions was confirmed. Interestingly, superposition of the *5AH0* and *1KU0* structures with that of LipJ 3D model provided evidence of the large overlapping between the three hydrolases (Fig 7B), indicating that they share a great structural homology. Moreover, putative Ca^2+^ and Zn^2+^-binding cavities [45,46,65,76] could also be predicted for LipJ after superposition of the three enzyme structures (Fig 7B), suggesting that LipJ could either be activated or dependent on such ions for activity. In fact, most described thermophilic and thermoalkaliphilic lipases bear either a Ca^2+^ or Zn^2+^-binding site, or both (seldom other ions; [12]), acting through a specific net of salt bridges [46,77] that have been shown to be essential for thermophilic activity and thermoresistance [21,65].

### Effect of metal ions on LipJ activity

**Fig 8:**
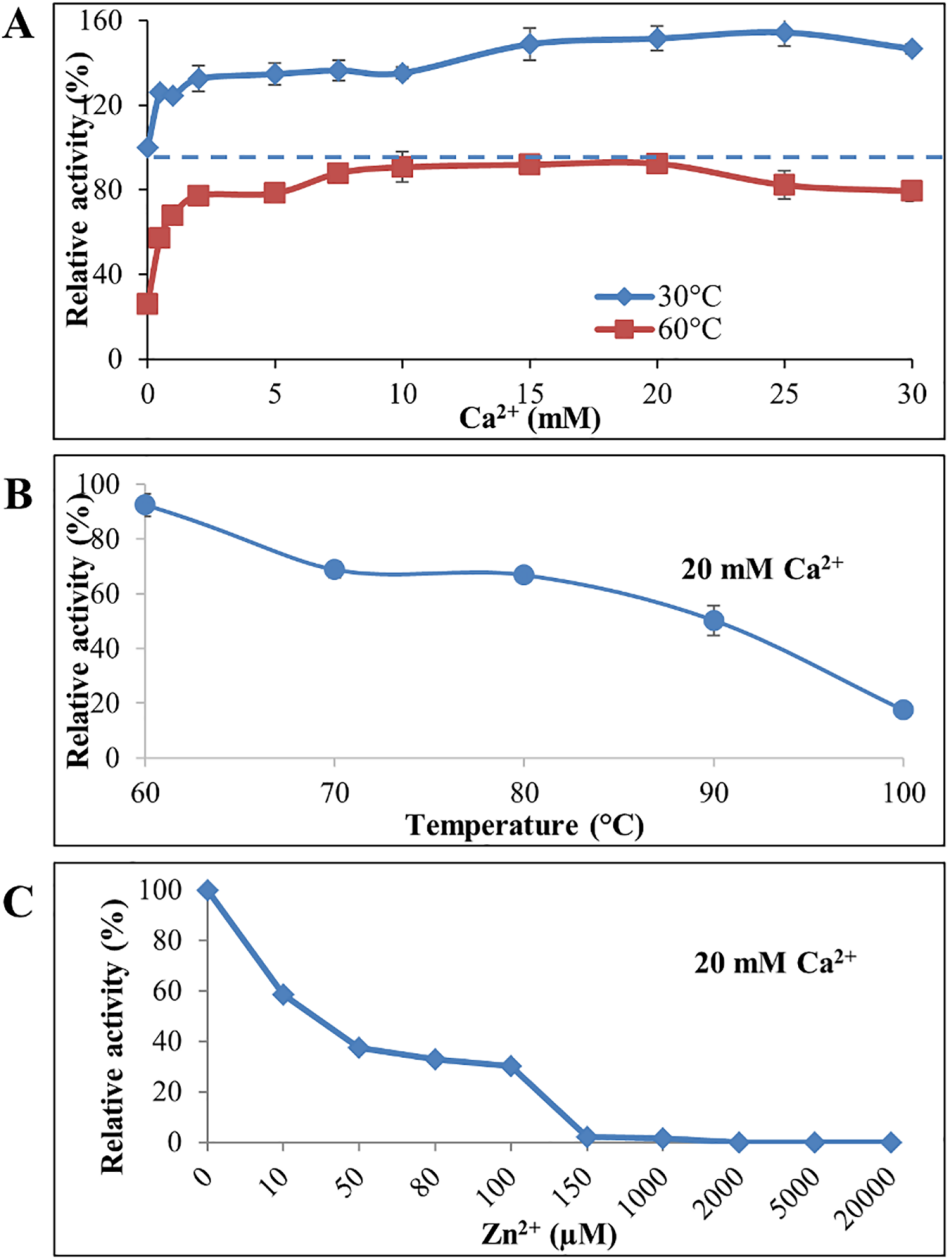
LipJ catalytic behaviour in the presence of Ca^2+^ and Zn^2+^ ions. (**A**) Activity of LipJ measured at 30 (blue diamonds) and 60°C (red squares) in the presence of different Ca^2+^ concentrations. (**B**) Activity of LipJ in the range of temperatures from 60 to 100°C in the presence of 20 mM Ca^2+^. (**C**) Loss of activity of LipJ assayed at 60°C in the presence of different Zn^2+^ concentrations.

From the previous *in silico* analysis it was shown that LipJ displays many traits of thermophilic lipases, including its phylogenetic relationship with typically thermophilic family I.5 lipases, the conserved structure and folding of thermophilic lipases, and the presence of putative cavities for accepting ions. Presence of such cavities could indicate that either Ca^2+^ or Zn^2+^ (or both) are required for activity at high temperature. This might justify the lack of activity shown by LipJ when assayed over 30°C in the absence of extra ions. To test this possibility, activity assays were carried out at 30 and 60°C in the presence of different concentrations of Ca^2+^ or Zn^2+^ or both, and other ions. As shown in Fig 8A, a substantial gain in LipJ activity at 60°C was achieved when the reaction mix was supplemented with Ca^2+^, obtaining maximum activity at 20 mM Ca^2+^. However, the highest activity values of LipJ at 60°C with Ca^2+^ were always lower than those found at 30°C with or without Ca^2+^, indicating that presence of this ion in the reaction mixture improves in fact the thermal range of LipJ but it does not shift the enzyme to a completely thermophilic behaviour. This was further confirmed when assays were performed in the presence of 20 mM Ca^2+^ in a range of temperatures from 60 to 100°C. Although an important loss of activity was observed at temperatures above 60°C, LipJ still retained some activity in a wider range of temperatures, showing approximately 65% and 20% residual activity when assayed for 15 minutes at 80 and 100°C, respectively (Fig 8B).

**Fig 9:**
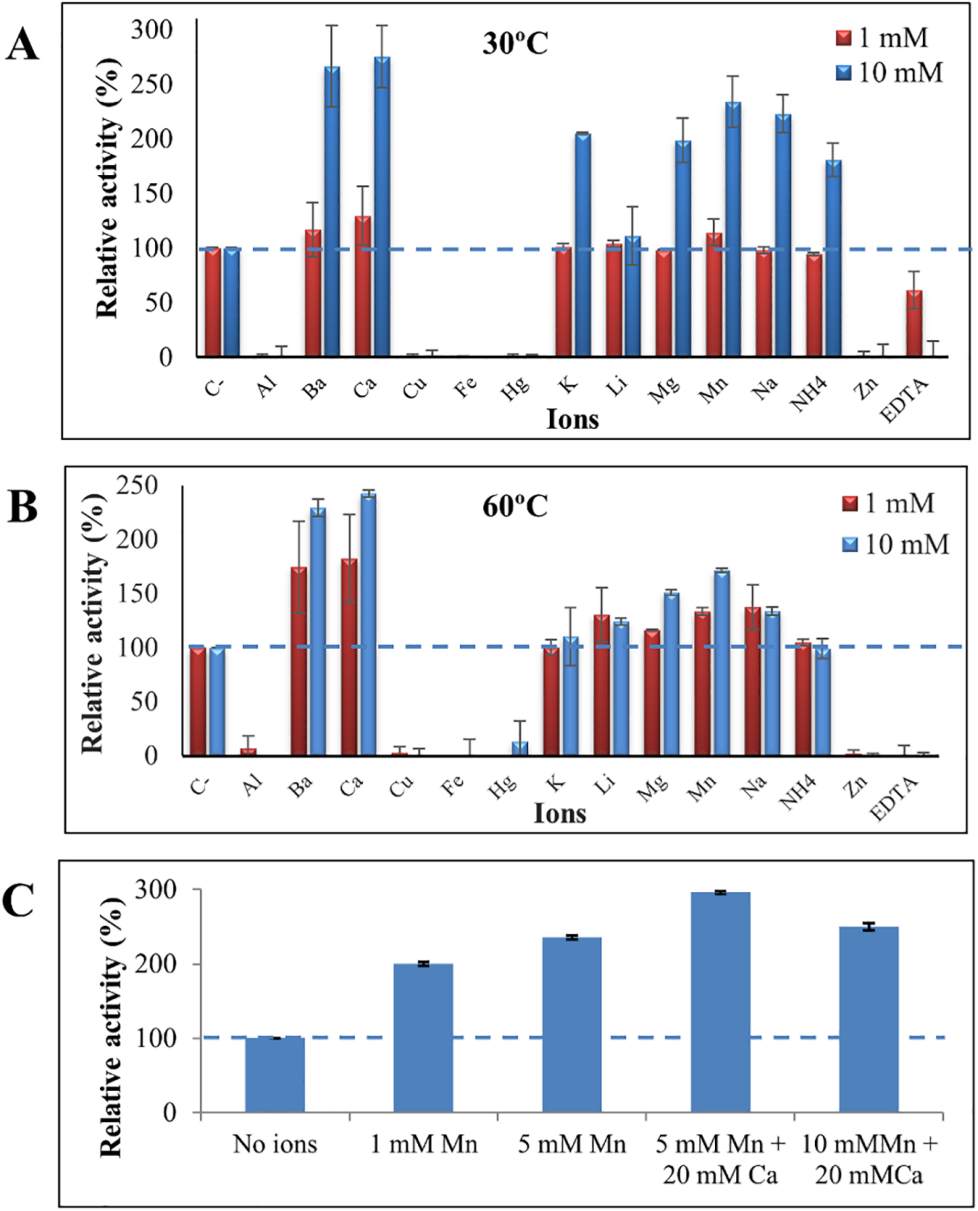
Effect of different metal ions (assayed at 1 and 10 mM) and EDTA on LipJ activity, measured at 30 (**A**) and 60°C (**B**). (**C**) Enhanced activity of LipJ by a combination of 20 mM Ca^2+^ and 5 mM Mn^2+^, assayed at 80°C.

When activity was tested at 60°C in the presence of different concentrations of Zn^2+^ or both, Ca^2+^ and Zn^2+^, complete loss of activity resulted in all cases at Zn^2+^ concentrations over 0.15 mM, showing only 40% activity at 0.05 mM Zn^2+^ with respect to that found without ions at 30°C (Fig 8C). When other ions and EDTA were assayed (Fig 9A and 9B), it was shown that Ba^2+^ and Mn^2+^ contribute also to increase activity at high temperatures. In fact, a 3-fold activity increase at 80°C was obtained with a combination of 20 mM Ca^2+^ and 5 mM Mn^2+^ (Fig 9C). However, neither the enhanced activity at high temperatures produced by Ca^2+^, nor that of Ba^2+^ and Mn^2+^ could completely restore the high activity at 100°C, indicating that cloned LipJ is most probably a different enzyme from that found in the supernatant of *Bacillus* JR3.

From the above results we conclude that although LipJ is not responsible for the thermophilic activity found in supernatants of strain JR3, it displays many features of thermophilic lipases like the close phylogenetic proximity to family I.5 lipases, the high similarity of the 3D model structure, plus the observation of lipolytic activity at higher temperatures with the same ions required by thermophilic lipases. Moreover, large amino acid signatures of thermophilic lipases are conserved in LipJ, which was cloned using degenerated primers designed for thermophilic lipases of related genera. Altogether, the results obtained here allow to hypothesize that LipJ could have been in fact a thermophilic extracellular lipase/esterase in ancestral times, when the environmental conditions were more extreme, having evolved to the present mesophilic profile. The change from extreme to mild conditions in the volcanic island of El Hierro could have prompted evolution of *Bacillus* JR3 towards a mesophilic strain (optimum growth temperature 30°C), yet retaining certain thermophilic traits like the secreted thermophilic lipase/esterase activity observed. In ancestral times, the extracellular thermophilic lipolytic system of *Bacillus* JR3 (range of temperatures from 30 to 100°C) could have been useful when search for nutrients was harsh and required the presence of duplicated specialised enzymes for survival. However, once the environmental conditions changed, mutations of the enzyme could have been positively selected for mesophilic adaptation, and LipJ (range of temperature from 15 to 40°C) could have evolved from a thermophilic scenario to adapt to the present mild, mesophilic conditions.

## Conclusions

We provide here evidence that the newly isolated strain *Bacillus* sp. JR3 from the Laurisilva Forest of El Hierro Island displays a complex thermophilic and thermostable extracellular lipolytic system, with great potential for industrial applications requiring biocatalysts adapted to harsh temperature conditions. PCR prospection of JR3 genome using degenerated consensus primers for thermophilic lipases allowed cloning and characterization of the new esterase LipJ. The cloned enzyme displays a mesophilic profile, showing preference for short chain-length substrates, with a kinetics profile typical of an esterase. Although being a member of the bacterial lipase family I.5 and bearing several traits of thermophilic lipases, identified in the amino acid sequence and the 3D structure model, LipJ is indeed a mesophilic enzyme. The mixture of thermophilic plus mesophilic features shown by LipJ allows to hypothesise that this enzyme could have evolved from a thermophilic lipase after adaptation to the present mild environment.
